# Special Notice

**Published:** 1877-10-20

**Authors:** 


SPECIAL NOTICE.—We wish to say to our subscribers, and to any others who may receive this number of our journal, that the correspondence of the office gives abundant evidence that the *practical character of* THE RECORD is attracting the attention of the profession, and that it meets a *desideratum* long felt by the busy practitioner. Further evidence also exists in the steady increase of our subscription list, and in the enlargement of our advertising department.

A change of type giving some addition to the amount of reading matter, and other improvements in THE RECORD, will be made at the beginning of the ensuing year, and no efforts will be spared to make it more and more interesting and useful to our readers. The present low price of subscription and club rates will be continued. It is hoped, therefore, that each one now on our list will not only renew his subscription, but will induce others to subscribe; and, in order to encourage them to begin the work at once, we will allow new subscribers for 1878 to commence with the November or December number of the present year. Friends, give us a lift!

